# Neonatal mortality rates in Brazilian municipalities: from 1996 to 2017

**DOI:** 10.1186/s13104-020-05441-3

**Published:** 2021-02-09

**Authors:** Lais Baroni, Rebecca Salles, Samella Salles, Marcel Pedroso, Jefferson Lima, Igor Morais, Lucas Carraro, Raphael de Freitas Saldanha, Carlos Sousa, Carlos Cardoso, Balthazar Paixño, Sérgio Cruz, Eduardo Ogasawara, Patrícia de Morais Mello Boccolini, Cristiano Siqueira Boccolini

**Affiliations:** 1 Federal Center for Technological Education of Rio de Janeiro, CEFET/RJ, Rio de Janeiro, Brazil; 2 Platform of Data Science Applied to Health (PCDaS), ICICT, Oswaldo Cruz Foundation, Fiocruz, Rio de Janeiro, Brazil; 3 Oswaldo Cruz Foundation, Fiocruz, Rio de Janeiro, Brazil; 4 Federal Institute of Rio de Janeiro, IFRJ, Rio de Janeiro, Brazil

**Keywords:** Neonatal mortality rate, Time series data, Brazilian municipalities

## Abstract

**Objectives:**

Neonatal mortality is a global public health problem, and the efforts to reduce child mortality is one of the goals of the 2030 Agenda for Sustainable Development, launched in 2015 by the United Nations. The availability of historical neonatal mortality rates (NMR) data in Brazilian municipalities is crucial to evaluate trends at local, regional and national level, identifying gaps and vulnerable territories. Therefore, the objective of this article is to offer an integrated dataset containing monthly data in a historical series from 1996 to 2017 with information on all births, neonatal deaths, and NMR (total, early and late components) enriched with information related to the municipality.

**Data description:**

It is a dataset of historical data with information on the number of births, the number of neonatal deaths, the neonatal mortality rate (including early and late), and geographic information for each month (between January 1996 and December 2017) and Brazilian municipality.

## Objectives

Neonatal mortality, defined as the number of deaths of children from 0 to 27 days of life per thousand live births, represents a global public health problem [1]. The neonatal mortality rate (NMR) has dropped in Brazil from 37 deaths per thousand live births in 1990 to 18 deaths per thousand live births in 2017, a significant reduction of 51%. Nonetheless, these values represent the deaths of 272,439 Brazilian newborns in the last 10 years [2–4].

Reducing neonatal mortality is an essential part of efforts to reduce child mortality and corresponds to a goal of the 2030 Agenda for Sustainable Development, launched in 2015 by the United Nations [5]. The third Sustainable Development Goal (SDG) aims to “end, by 2030, preventable deaths of newborns and children under 5 years of age, with all countries aiming to reduce neonatal mortality to at least as low as 12 deaths per 1000 live births and under-5 mortality to at least as low as 25 deaths per 1000 live births”.

In the epidemiology, the process of knowledge discovery in databases (KDD) can assist in the determination of public policies to be implemented, such as improving neonatal care or adopting pro-breastfeeding techniques [6]. The datasets presented in this document are directed precisely at this objective. They can easily be integrated with additional information such as population density, human development index (HDI), quality of prenatal care, among others. Therefore, this work’s main contribution is to provide a consolidated historical datasets with information related to neonatal mortality in Brazilian municipalities.

## Data description

Since mothers can live in one municipality and have delivery in another one, in this work, two datasets built analogously containing the same attributes are available: $NM\_County\_B$ and $NM\_County\_R$. While in $NM\_County\_B$, the instances correspond to the municipality of birth of the neonates, in $NM\_County\_R$, the instances correspond to the municipality where the neonate’s mother lives, both for each available competency date. These datasets and related files are available for download, as shown in Table 1.

**Table 1:** Overview of data files/datasets

Label	Name of data file/dataset	File type (file extension)	Data repository and identifier
Dataset 1	NM_County_B	Delimited text (.csv)	Synapse: https://doi.org/10.7303/syn22241002 [7]
Dataset 2	NM_County_R	Delimited text (.csv)	Synapse: https://doi.org/10.7303/syn22241002 [7]
Data file 1	Dict_NM_county	Delimited text (.csv)	Synapse: https://doi.org/10.7303/syn22241002 [7]
Data file 2	Data_construction	Portable document (.pdf)	Synapse: https://doi.org/10.7303/syn22241002 [7]
Data file 3	Data_validation	R code (.r)	Synapse: https://doi.org/10.7303/syn22241002 [7]
Data file 4	Municipalities_codes_IBGE	Delimited text (.csv)	Synapse: https://doi.org/10.7303/syn22241002 [7]
Data file 5	Data_construction	Jupyter notebook (.ipynb)	Synapse: https://doi.org/10.7303/syn22241002 [7]
Data file 6	ETLTMNOCOR	Dataiku DSS project (.zip)	Synapse: https://doi.org/10.7303/syn22241002 [7]
Data file 7	ETLTMNRES	Dataiku DSS project (.zip)	Synapse: https://doi.org/10.7303/syn22241002 [7]
Data file 8	Data_translation	Jupyter notebook (.ipynb)	Synapse: https://doi.org/10.7303/syn22241002 [7]

### Data construction

The datasets developed are the result of a construction process organized into three main activities: (i) data integration, (ii) creation of neonatal mortality rates, and (iii) data enrichment. The criteria adopted for data management were based on detailed studies of the dataset and the support of specialists in the field.^1^

i. The developed datasets integrate data from the Live Birth Information System (SINASC) and the Mortality Information System (SIM). Both are made available by DATASUS and represent individual records of live births and deaths. Such records were aggregated by competency date (month and year) and municipality (place of birth and mother’s residence), creating absolute values with the total number of births and deaths. SIM data were filtered, selecting only neonatal deaths, and then separated into general (0–27 days), early (0–7 days) and late (8–27 days) neonatal deaths.
ii Other temporal aggregations (semiannual and annual moving average) and spatial aggregations (states, regions, and country) were constructed from the absolute values of births and neonatal deaths. Then, the general, early, and late neonatal mortality rates (NMR) were calculated for each instance aggregated in time (month, semiannual, and annual moving average) and space (municipalities, states, regions, and country) according to Eq. 1.
  1 $$\begin{aligned} \text {NMR} = \frac{\text {number of neonatal deaths}}{\text {number of births}} \cdot 1000 \end{aligned}.$$
iii. The data was enriched with information about the municipalities, states, and regions obtained from the DATASUS FTP server.^2^ The attributes resulting from this process are the coordinates, area, regional health codes, and Boolean attributes, indicating if the municipality is the state’s capital, border. The data dictionary^3^ presents a full description.

### Data validation

The datasets $NM\_County\_B$ and $NM\_County\_R$ present, respectively, 845,561 and 1,413,773 records and 106 attributes. There are 36 attributes regarding the number of deaths. There is an attribute for each combination in time (month, moving average, and year), space (municipality, federation state, region, and country), and type of neonatal death (general, late, and early). Thirty-six attributes associated with rates also exist, analogous to those of deaths. The attributes regarding births differ due to not having the distinction of the type of delivery and. Therefore, they are 24. There are 18 attributes with information about the municipalities. There are still the attributes year, month, and year/month in timestamp format.^4^

In $NM\_County\_B$, approximately 66% of the municipalities have more than half of the competency dates, and a little more than 44% have more than 90%. In $NM\_County\_R$, almost all municipalities (99.8%) have more than half of the competency dates, and a little more than 88% have more than 90%. $NM\_County\_B$ includes 4998, and $NM\_County\_R$ comprises all municipalities of the 5570 existing in Brazil. The differences among two datasets exists due not all municipalities in Brazil have birth delivery facilities or maternity wings, forcing mothers to have their babies in surrounding cities.

## Limitations

- Some municipalities do not have a complete historical series and may only have data corresponding to a short period or with very sparse intervals. This happens because 68% of Brazilian municipalities had less than 20 thousand inhabitants, increasing the number of months without births, and subsequent absence of NMR.
- There are cases where the rates exceed one thousand, that represents more deaths than births. These results may reflect the death of a baby in a month without births. We suggest, during analysis, the addition of a filter to ignore the NMR above 200/1000 births.
- $NM\_County\_B$ does not include all municipalities of the country since not all have maternity hospital or birth delivery facilities.
- Since neonatal deaths are a relatively rare event, and many of Brazilian municipalities have populations with less than 20,000 inhabitants, for the vast majority of competency dates, the number of neonatal deaths in the municipality is equal to zero. It makes the corresponding $NM\_County\_R$ also equal to zero. In $NM\_County\_B$, 81.13% of the records have a monthly $NM\_County\_R$ equal to zero. In $NM\_County\_R$, the percentage is 78.21%.
- The municipalities of Aroeiras do Itaim, Figueirão, Ipiranga do Norte, and Itanhangá were created on January 1st, 2005. Therefore, it was not possible to find information on coordinates and area for them. These are the only empty fields (NAs) in the datasets.

## Data Availability

The dataset generated during the current study and additional documentation is freely and openly available on the Synapse repository at https://doi.org/10.7303/syn22241002 [7].

## Abbreviations

NMR: Neonatal mortality rate
DATASUS: Department of Informatics of the Unified Health System of Brazil
SUS: Unified Health System
SINASC: Brazil Live Birth Information System
SIM: Brazil Mortality Information System
KDD: Knowledge Discovery in Databases

## Footnotes

1 Description of the construction of the datasets is available at https://www.synapse.org/#!Synapse:syn22288740

2 Available at ftp://ftp.datasus.gov.br/dissemin/publicos/SINASC/NOV/TABELAS/.

3 Available at https://www.synapse.org/#!Synapse:syn22241065.

4 The timestamp format represents the seconds since the date of origin, 01/01/1970.
